# Supplementary material for: Quality by Design-Based Development of Solid Self-Emulsifying Drug Delivery System (SEDDS) as a Potential Carrier for Oral Delivery of Lysozyme
Source: Pharmaceutics. 2023 Mar 20;15(3):995. doi: 10.3390/pharmaceutics15030995 (PMC10058933; doi:10.3390/pharmaceutics15030995)
Supplement: Supplementary file 1 [file pharmaceutics-15-00995-s001.zip › pharmaceutics-2247780-supplementary.pdf]

SUPPLEMENTARY MATERIAL

Table S1. Design matrix for factor screening as per two-level five-factor fractional factorial design (2^{5-2}).

Experiment	*Molar ratio (LYS:SDS)* X_1	*pH* X_2	*Mixing speed (rpm)* X_3	*Mixing time (min.)* X_4	*Temperature (°C)* X_5
1	1:2	4	300	30	37
2	1:6	4	300	15	25
3	1:2	9	300	15	37
4	1:6	9	300	15	25
5	1:2	4	600	30	25
6	1:6	4	600	30	37
7	1:2	9	600	15	25
8	1:6	9	600	30	37

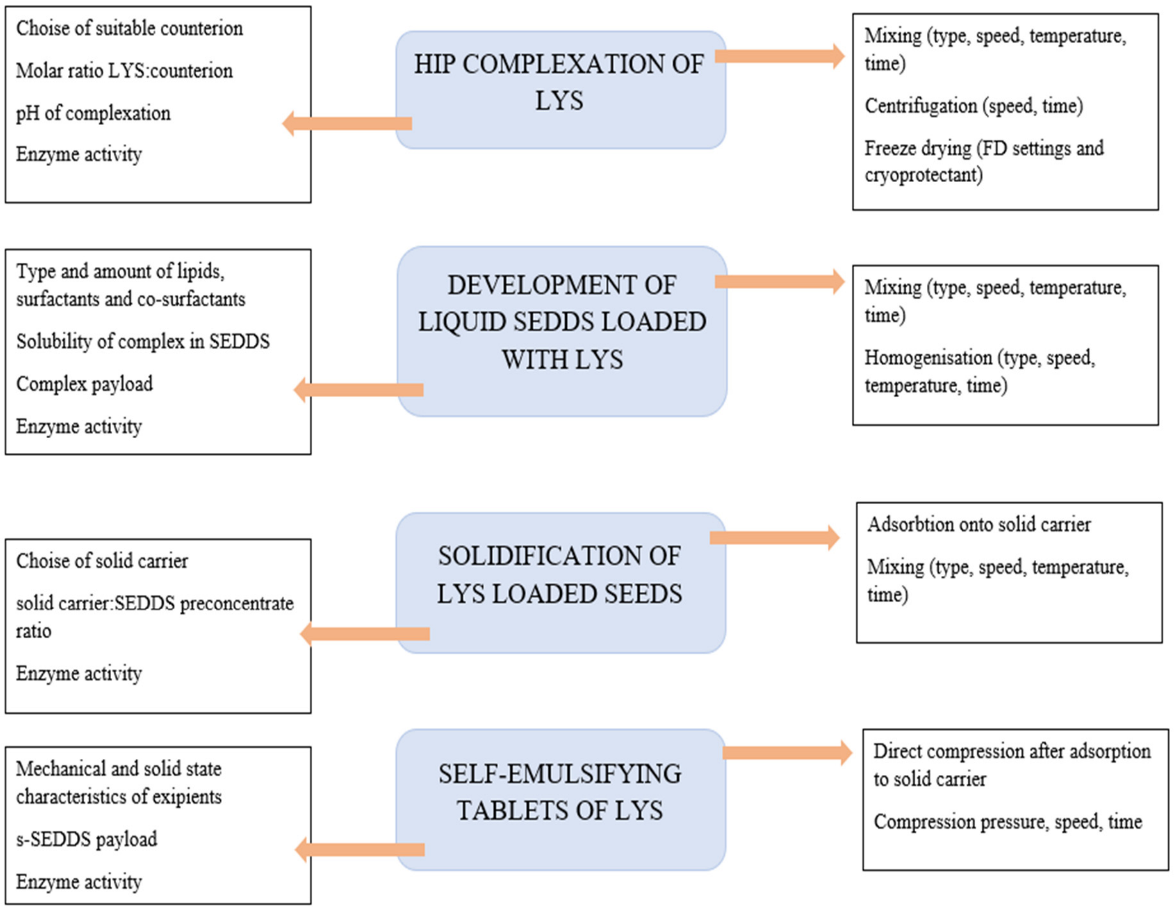

Figure S1. Flow chart of the development process.

Table S2. Interdependence rating between CMAs/CPPs, and CQAs -liquid SEDDS.

CQA	CMAs/CPPs							
	Amount of lipid	Amount of surfactant	Amount of co-surfactant	Mixing type	Mixing speed	Mixing temperature	Mixing time	pH and type of dilution media
Droplet size	High	High	High	Medium	Medium	Medium	High	High
Zeta potential	High	Medium	High	Medium	Low	Low	Low	High
PdI	High	High	High	Medium	Medium	Medium	High	High
Emulsification efficiency	High	High	High	Medium	Medium	Medium	High	High
HIPs payload	Medium	High	High	Low	Low	Low	Low	High
Release profile	High	High	High	Medium	Medium	Medium	Medium	Medium
Permeability	High	High	High	Low	Low	Low	Low	Medium
Enzyme sensitivity	Medium	High	Medium	Medium	Medium	Medium	Medium	High

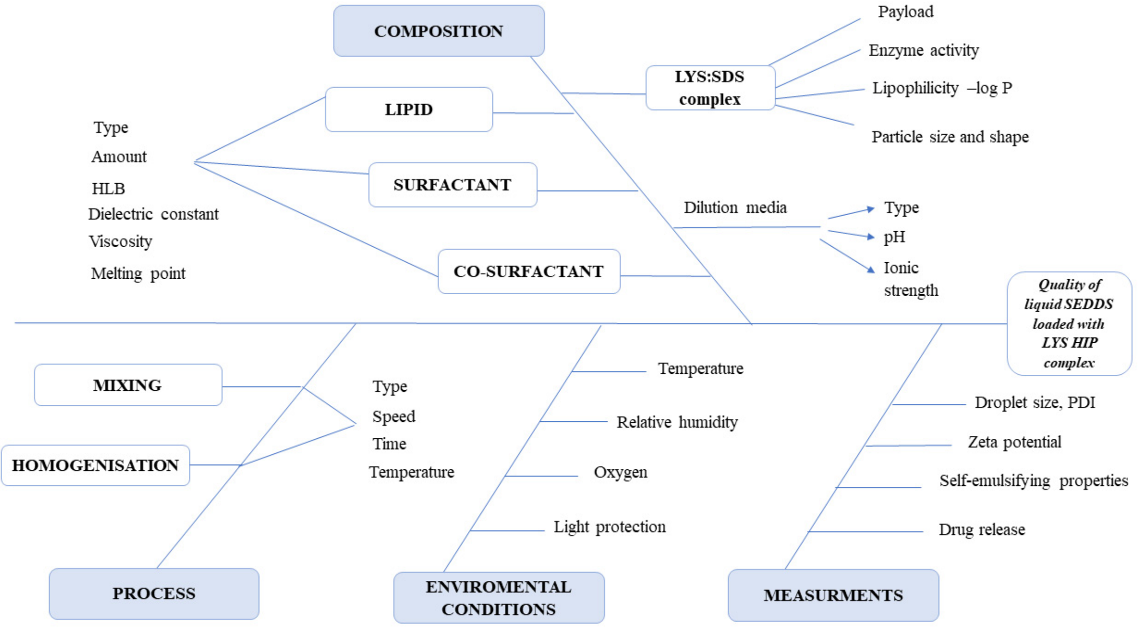

Figure S2. Ishikawa diagram -the quality of liquid SEDDS loaded with LYS HIP complex.

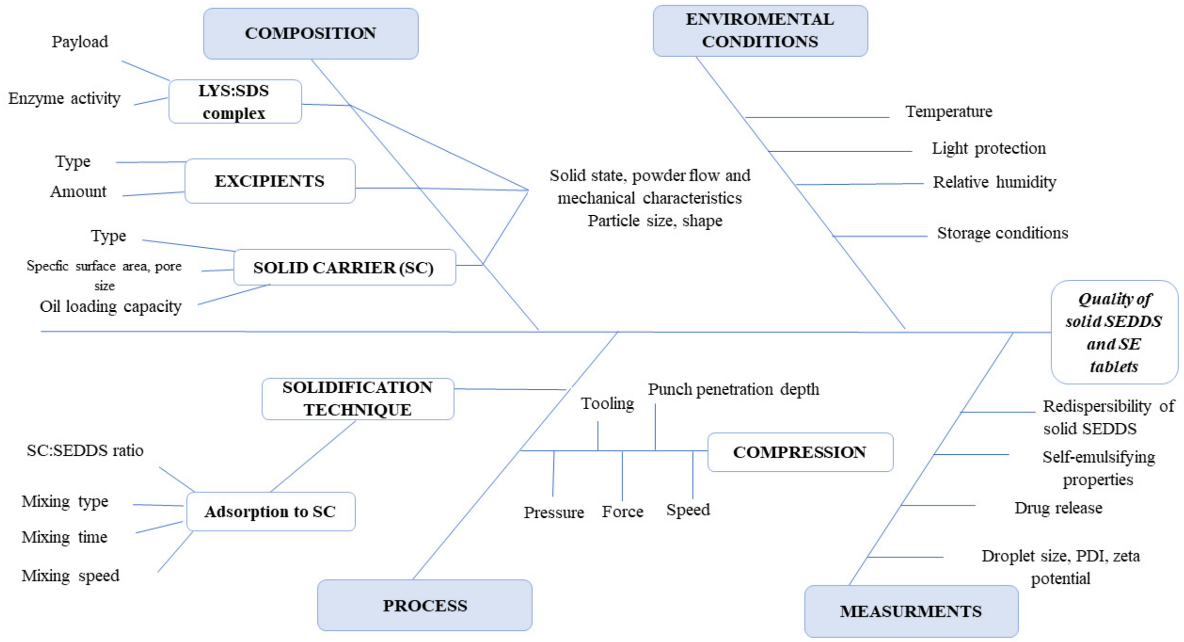

Figure S3. Ishikawa diagram -the quality of solid SEDDS loaded with LYS HIP complex.

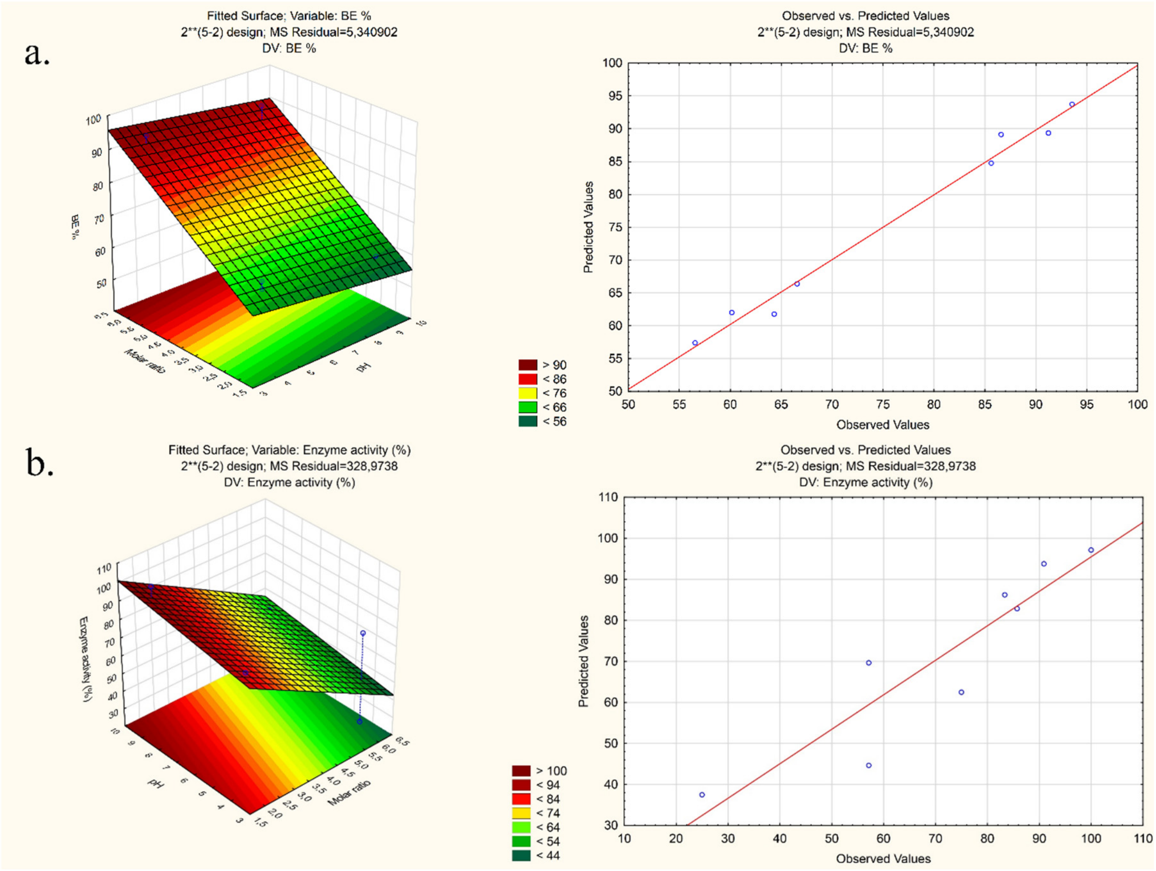

Figure S4. Two-level five-factor fractional factorial design (2^{5-2}); the response surfaces showing the effect of the significant examined variables on the responses and observed vs. predicted values of (a) binding efficiency (b) enzyme activity.

Table S3. Two-level five-factor fractional factorial design (2^{5-2})-detail statistical results.

Factor	Effect Estimates; Var.:BE %; R-sqr=,98664; Adj:,97662 (Design: 2**(5-2) design (Spreadsheet1) in Workbook2) 2**(5-2) design; MS Residual=5,340902 DV: BE %									
	Effect	Std.Err.	t(4)	p	-95,% (Cnf.Limt)	+95,% (Cnf.Limt)	Coeff.	Std.Err. (Coeff.)	-95,% (Cnf.Limt)	+95,% (Cnf.Limt)
Mean/Interc.	75.56912	0.817076	92.48729	0.000000	73.30055	77.83768	75.56912	0.817076	73.30055	77.83768
(1)Molar ratio	27.35984	1.634151	16.74253	0.000075	22.82271	31.89697	13.67992	0.817076	11.41135	15.94848
(5)Temperature	-4.60709	1.634151	-2.81925	0.047864	-9.14422	-0.06996	-2.30354	0.817076	-4.57211	-0.03498
(2)pH	-4.37424	1.634151	-2.67676	0.055415	-8.91137	0.16289	-2.18712	0.817076	-4.45568	0.08145

Factor	Effect Estimates; Var.:Enzyme activity; R-sqr=,83843; Adj:,62301 (Design: 2**(5-2) design)2**(5-2) design; MS Residual=221,7099 DV: Enzyme activity									
	Effect	Std.Err.	t(3)	p	-95,% (Cnf.Limt)	+95,% (Cnf.Limt)	Coeff.	Std.Err. (Coeff.)	-95,% (Cnf.Limt)	+95,% (Cnf.Limt)
Mean/Interc.	71,7775	5,26438	13,63455	0,000853	55,0239	88,53112	71,7775	5,264384	55,0239	88,53112
(1)Molar ratio	-36,4150	10,52877	-3,45862	0,040681	-69,9222	-2,90776	-18,2075	5,264384	-34,9611	-1,45388
(3)Mixing speed	-14,1800	10,52877	-1,34679	0,270755	-47,6872	19,32724	-7,0900	5,264384	-23,8436	9,66362
(5)Temperature	-10,8200	10,52877	-1,02766	0,379722	-44,3272	22,68724	-5,4100	5,264384	-22,1636	11,34362
(2)pH	9,0350	10,52877	0,85813	0,453915	-24,4722	42,54224	4,5175	5,264384	-12,2361	21,27112

Table S4. 2^3 full factorial design -detail statistical results.

Factor	Effect Estimates; Var.:Binding efficiency (%); R-sqr=,96138; Adj:,92275 (Design: 2**(3-0) design 2**(3-0) design; MS Residual=13,05386 DV: Binding efficiency (%)									
	Effect	Std.Err.	t(2)	p	-95,% (Cnf.Limt)	+95,% (Cnf.Limt)	Coeff.	Std.Err. (Coeff.)	-95,% (Cnf.Limt)	+95,% (Cnf.Limt)
Mean/Interc.	74.73111	1.7028	43.88642	0.0005	67.4044251	82.05780	74.73111	1.7028	67.40443	82.05780
(1)Molar ratio	25.46000	3.6122	7.04824	0.0195	9.91775181	41.00225	12.73000	1.8061	4.95888	20.50112
1 by 3	-1.07000	3.6122	-0.29621	0.7950	-16.6122482	14.47225	-0.53500	1.8061	-8.30612	7.23612
1*2*3	0.57000	3.6122	0.15780	0.8891	-14.9722482	16.11225	0.28500	1.8061	-7.48612	8.05612
2 by 3	-0.42500	3.6122	-0.11766	0.9171	-15.9672482	15.11725	-0.21250	1.8061	-7.98362	7.55862
(3)Temperature	0.09500	3.6122	0.02630	0.9814	-15.4472482	15.63725	0.04750	1.8061	-7.72362	7.81862
(2)pH	0.04500	3.6122	0.01246	0.9912	-15.4972482	15.58725	0.02250	1.8061	-7.74862	7.79362
Factor	Effect Estimates; Var.:Enzyme activity (%); R-sqr=,98673; Adj:,96461 (Design: 2**(3-0) design 2**(3-0) design; MS Residual=8,458545 DV: Enzyme activity (%)									
	Effect	Std.Err.	t(2)	p	-95,% (Cnf.Limt)	+95,% (Cnf.Limt)	Coeff.	Std.Err. (Coeff.)	-95,% (Cnf.Limt)	+95,% (Cnf.Limt)
Mean/Interc.	81.7518	1.1682372	69.978749	0.00020414	76.7253	86.7782968	81.7517778	1.1682372	76.7253	86.7782968
(1)Molar ratio	-19.5163	2.47820534	-7.87515453	0.01574454	-30.1791	-8.85339303	-9.758125	1.23910267	-15.0896	-4.42669652
1 by 2	15.5653	2.47820534	6.28085565	0.02442422	4.9024	26.228107	7.782625	1.23910267	2.4512	13.1140535
(2)pH	14.9113	2.47820534	6.016955	0.02652729	4.2484	25.574107	7.455625	1.23910267	2.1242	12.7870535
(3)Temperature	9.5423	2.47820534	3.85046786	0.06131099	-1.1206	20.205107	4.771125	1.23910267	-0.5603	10.1025535
2 by 3	2.6098	2.47820534	1.05308061	0.4027547	-8.0531	13.272607	1.304875	1.23910267	-4.0266	6.63630348
1 by 3	0.6363	2.47820534	0.25673821	0.82137825	-10.0266	11.299107	0.318125	1.23910267	-5.0133	5.64955348

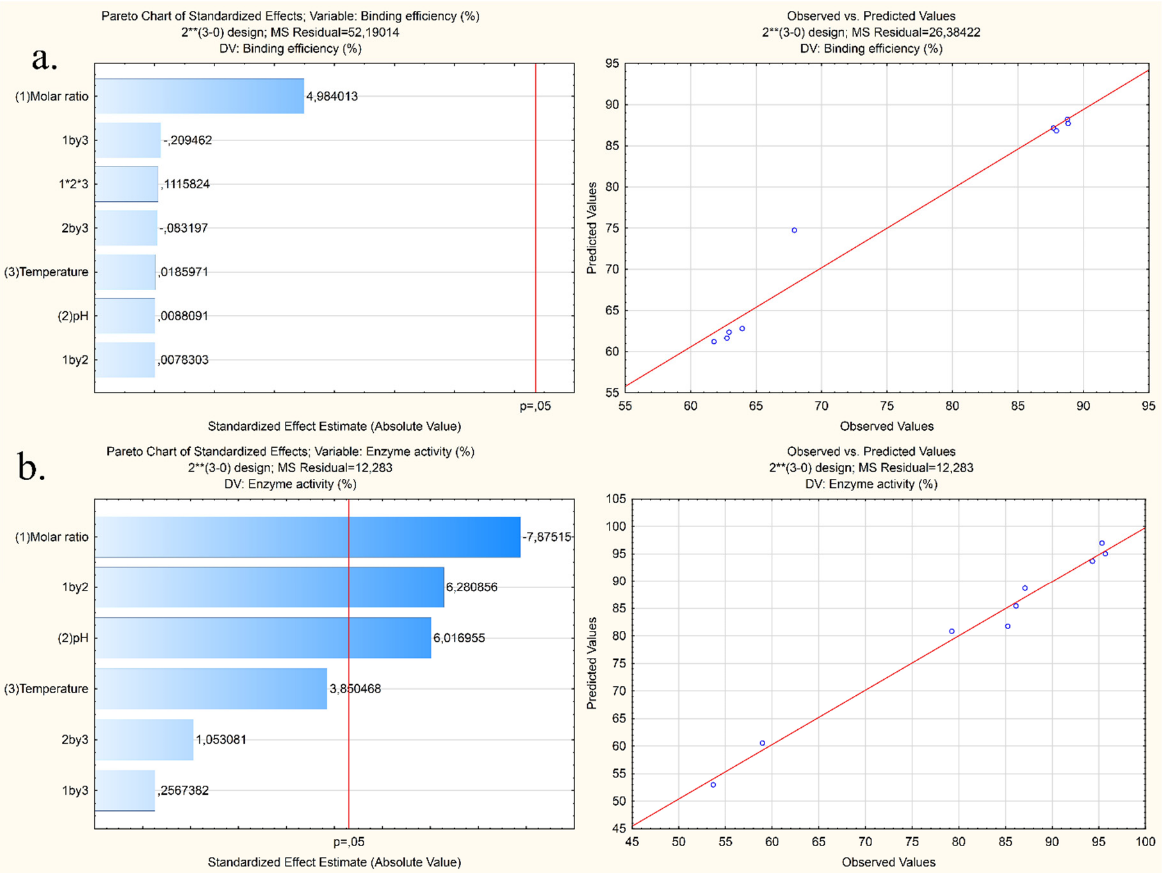

Figure S5. Pareto charts of the effects and predicted values of (a) binding efficiency (b) enzyme activity.

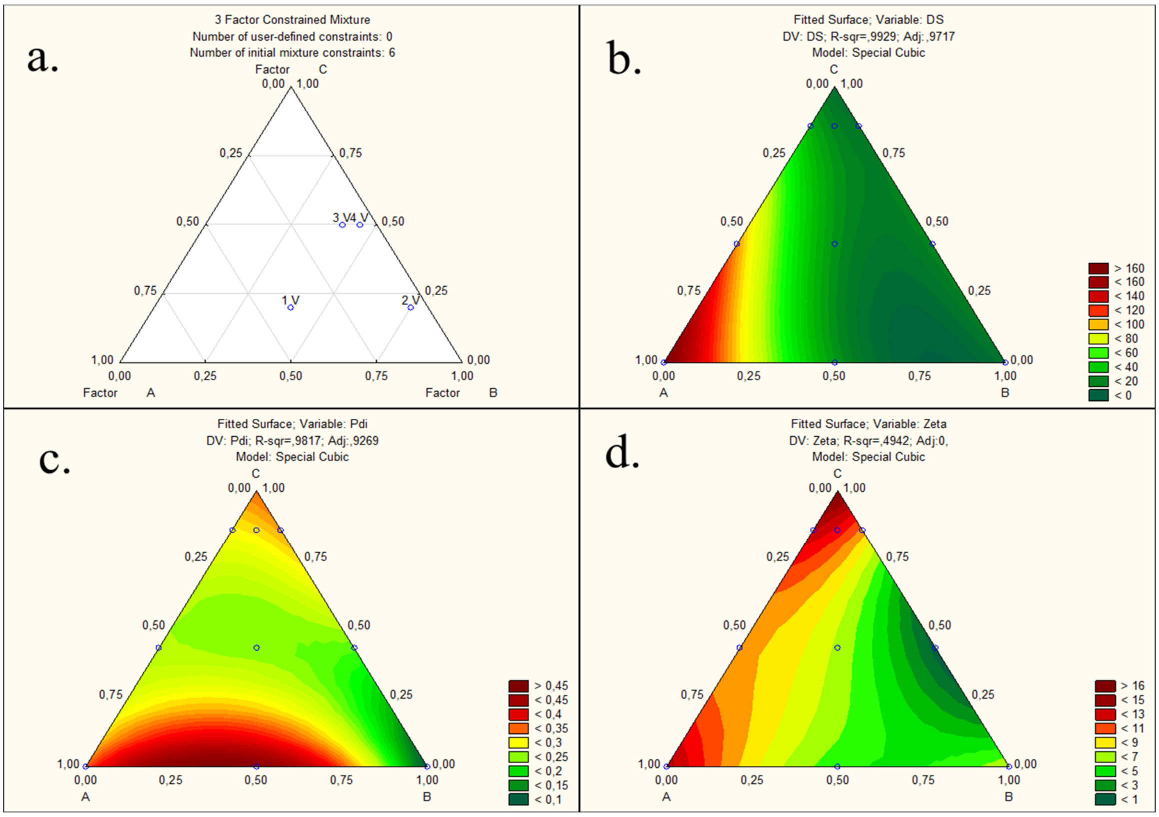

Figure S6. (a) 3 factor constrained mixture design; two-dimensional contour plots of (b) droplet size (c) PDI (d) zeta potential.

Table S5. Mixture design-detail statistical results.

Factor	Coeffs (recoded comps); Var.:DS; R-sqr=,9929; Adj:,9717 (3 Factor Constrained Mixture (3 Factor Constrained Mixture ([No active dataset]) in Constrained mixtures) in Constrained mixtures) 3 Factor mixture design; Mixture total=100,, 9 Runs DV: DS; MS Residual=92,81305					
Factor	Coeff.	Std.Err.	t(2)	p	-95,%	+95,%
(A)A	178.187	9.5949	18.57108	0.002887	136.90	219.470
(B)B	10.383	9.5949	1.08214	0.392307	-30.90	51.666
(C)C	6.220	10.0419	0.61938	0.598823	-36.99	49.427
AB	-280.019	47.1869	-5.93425	0.027242	-483.05	-76.990
AC	-2.723	51.9775	-0.05240	0.962976	-226.36	220.918
BC	40.850	51.9775	0.78592	0.514240	-182.79	264.491
ABC	-447.191	352.0373	-1.27029	0.331762	-1961.89	1067.503
	Coeffs (recoded comps); Var.:DS; R-sqr=,9929; Adj:,9811 (3 Factor Constrained Mixture (3 Factor Constrained Mixture ([No active dataset]) in Constrained mixtures) in Constrained mixtures) 3 Factor mixture design; Mixture total=100,, 9 Runs DV: DS; MS Residual=61,9603					
Factor	Coeff.	Std.Err.	t(3)	p	-95,%	+95,%
(A)A	177.964	7.0267	25.32689	0.000135	155.60	200.326
(B)B	10.353	7.8259	1.32296	0.277649	-14.55	35.259
(C)C	5.883	6.3072	0.93278	0.419740	-14.19	25.955
AB	-279.522	37.7693	-7.40078	0.005103	-399.72	-159.324
BC	41.920	39.0572	1.07329	0.361793	-82.38	166.217
ABC	-455.060	260.1446	-1.74926	0.178559	-1282.96	372.836
	Coeffs (recoded comps); Var.:Pdi; R-sqr=,9817; Adj:,9269 (3 Factor Constrained Mixture (3 Factor Constrained Mixture ([No active dataset]) in Constrained mixtures) in Constrained mixtures) 3 Factor mixture design; Mixture total=100,, 9 Runs DV: Pdi; MS Residual=,0007926					
Factor	Coeff.	Std.Err.	t(2)	p	-95,%	+95,%
(A)A	0.34727	0.028039	12.38524	0.006456	0.22663	0.467907
(B)B	0.09636	0.028039	3.43677	0.075234	-0.02428	0.217004
(C)C	0.34349	0.029345	11.70518	0.007220	0.21723	0.469752
AB	1.03423	0.137893	7.50022	0.017316	0.44092	1.627530
AC	-0.38345	0.151892	-2.52448	0.127568	-1.03699	0.270090
BC	0.12711	0.151892	0.83687	0.490728	-0.52642	0.780654
ABC	-2.40518	1.028748	-2.33797	0.144358	-6.83153	2.021161
	Coeffs (recoded comps); Var.:Pdi; R-sqr=,9753; Adj:,9342 (3 Factor Constrained Mixture (3 Factor Constrained Mixture ([No active dataset]) in Constrained mixtures) in Constrained mixtures) 3 Factor mixture design; Mixture total=100,, 9 Runs DV: Pdi; MS Residual=,0007134					
Factor	Coeff.	Std.Err.	t(3)	p	-95,%	+95,%
(A)A	0.34865	0.026555	13.12904	0.000954	0.26414	0.433159
(B)B	0.10677	0.023843	4.47787	0.020764	0.03089	0.182648
(C)C	0.35920	0.021402	16.78342	0.000461	0.29109	0.427308

AB	1.01106	0.128161	7.88893	0.004245	0.60319	1.418923
AC	-0.43337	0.132531	-3.26991	0.046780	-0.85514	-0.011591
ABC	-2.03789	0.882740	-2.30859	0.104165	-4.84716	0.771387
	Coeffs (recoded comps); Var.:Zeta; R-sqr=,4942; Adj:0, (3 Factor Constrained Mixture (3 Factor Constrained Mixture ([No active dataset]) in Constrained mixtures) in Constrained mixtures) 3 Factor mixture design; Mixture total=100,, 9 Runs DV: Zeta; MS Residual=69,48256					
Factor	Coeff.	Std.Err.	t(2)	p	-95,%	+95,%
(A)A	13.6188	8.3018	1.640465	0.242595	-22.10	49.339
(B)B	6.9348	8.3018	0.835335	0.491423	-28.78	42.654
(C)C	16.0176	8.6886	1.843516	0.206571	-21.37	53.402
AB	-19.4014	40.8277	-0.475202	0.681482	-195.07	156.266
AC	-21.9548	44.9727	-0.488179	0.673699	-215.46	171.547
BC	-41.9080	44.9727	-0.931854	0.449786	-235.41	151.594
ABC	97.5327	304.5945	0.320205	0.779171	-1213.03	1408.097
	Coeffs (recoded comps); Var.:Zeta; R-sqr=,3962; Adj:,0339 (3 Factor Constrained Mixture (3 Factor Constrained Mixture ([No active dataset]) in Constrained mixtures) in Constrained mixtures) 3 Factor mixture design; Mixture total=100,, 9 Runs DV: Zeta; MS Residual=33,1751					
Factor	Coeff.	Std.Err.	t(5)	p	-95,%	+95,%
(A)A	10.8101	4.70099	2.29953	0.069813	-1.274	22.89438
(B)B	5.3952	5.23042	1.03151	0.349592	-8.050	18.84044
(C)C	13.4962	4.60068	2.93354	0.032499	1.670	25.32266
BC	-30.8114	27.10625	-1.13669	0.307192	-100.490	38.86738

Table S6. Properties of investigated solid carriers and their ability to adsorb optimal liquid SEDDS formulation.

Solid carrier (SC)	*Characteristics (according to manufacturer)*	*Ratio (SC:SEDDS)*	*Visual observation*	*Max. SEDDS loading capacity (g/100g SC)*
Neusilin® UFL2	PS: 3.1 μm<br>Specific surface area (m2/g): 300<br>Oil adsorbing capacity (ml/g): 2.7 - 3.4	1:1	Good ✓	300.0
		1:2	Good ✓	
		1:3	Sticky powder ✓	
		1:4	Poor, overwetted	
Syloid® AL-1 FP	PS: 6.5–8.1 μm<br>Specific surface area (m2/g): 675<br>Oil adsorbing capacity (g/g): 80	1:1	Sticky powder, greasy appearance✓	100.0
		1:2	Poor, overwetted	
		1:3	Poor, overwetted	
Syloid® 244 FP	PS: 2.3–3.7 μm<br>Specific surface area (m²/g): 310<br>Oil adsorbing capacity (g/g): 300	1:1	Good ✓	200.0
		1:2	Sticky powder ✓	
		1:3	Sticky powder ✓	
